# Integrative Neuroinformatics for Precision Prognostication and Personalized Therapeutics in Moderate and Severe Traumatic Brain Injury

**DOI:** 10.3389/fneur.2021.729184

**Published:** 2021-09-07

**Authors:** Frederick A. Zeiler, Yasser Iturria-Medina, Eric P. Thelin, Alwyn Gomez, Jai J. Shankar, Ji Hyun Ko, Chase R. Figley, Galen E. B. Wright, Chris M. Anderson

**Affiliations:** ^1^Section of Neurosurgery, Department of Surgery, Rady Faculty of Health Sciences, University of Manitoba, Winnipeg, MB, Canada; ^2^Department of Human Anatomy and Cell Science, Rady Faculty of Health Sciences, University of Manitoba, Winnipeg, MB, Canada; ^3^Biomedical Engineering, Faculty of Engineering, University of Manitoba, Winnipeg, MB, Canada; ^4^Centre on Aging, University of Manitoba, Winnipeg, MB, Canada; ^5^Division of Anaesthesia, Department of Medicine, Addenbrooke's Hospital, University of Cambridge, Cambridge, United Kingdom; ^6^Department of Neurology and Neurosurgery, Faculty of Medicine, McGill University, Montreal, QC, Canada; ^7^McConnell Brain Imaging Centre, Montreal Neurological Institute, Montreal, QC, Canada; ^8^Ludmer Centre for Neuroinformatics and Mental Health, Montreal, QC, Canada; ^9^Department of Clinical Neuroscience, Karolinska Institutet, Stockholm, Sweden; ^10^Department of Neurology, Karolinska University Hospital, Stockholm, Sweden; ^11^Department of Radiology, Rady Faculty of Health Sciences, University of Manitoba, Winnipeg, MB, Canada; ^12^Neuroscience Research Program, Kleysen Institute for Advanced Medicine, Winnipeg, MB, Canada; ^13^Department of Pharmacology and Therapeutics, Rady Faculty of Health Sciences, University of Manitoba, Winnipeg, MB, Canada

**Keywords:** multi-modal data, neuroinformatics, precision medicine, traumatic brain injury, big data

## Abstract

Despite changes in guideline-based management of moderate/severe traumatic brain injury (TBI) over the preceding decades, little impact on mortality and morbidity have been seen. This argues against the “one-treatment fits all” approach to such management strategies. With this, some preliminary advances in the area of personalized medicine in TBI care have displayed promising results. However, to continue transitioning toward individually-tailored care, we require integration of complex “-omics” data sets. The past few decades have seen dramatic increases in the volume of complex multi-modal data in moderate and severe TBI care. Such data includes serial high-fidelity multi-modal characterization of the cerebral physiome, serum/cerebrospinal fluid proteomics, admission genetic profiles, and serial advanced neuroimaging modalities. Integrating these complex and serially obtained data sets, with patient baseline demographics, treatment information and clinical outcomes over time, can be a daunting task for the treating clinician. Within this review, we highlight the current status of such multi-modal omics data sets in moderate/severe TBI, current limitations to the utilization of such data, and a potential path forward through employing integrative neuroinformatic approaches, which are applied in other neuropathologies. Such advances are positioned to facilitate the transition to precision prognostication and inform a top-down approach to the development of personalized therapeutics in moderate/severe TBI.

## Background and Current State

Traumatic brain injury (TBI) is one of the leading causes of death and disability globally, carrying significant societal costs (1, 2). Though across the severity spectrum mild TBI is the most prevalent, moderate and severe TBI carry the highest mortality and morbidity burden per case (1, 3). The cornerstone of the clinical management of moderate/severe TBI relies on rapid access to tertiary neurosurgical and neurocritical care services, with the goal of reducing injury insult burden. The primary injury in TBI refers to the structural damage incurred at the time of injury and relies on public health level changes aimed at prevention and risk reduction. Thus, the primary injury burden cannot be changed by the treating clinician, other than policy-level engagements aimed at population-level incidence reduction strategies (1). However, secondary injury refers to the cascade of host cellular responses to the primary injury, which exposes the brain to ongoing insult over the acute and subacute phases of care. The secondary injury cascade is theoretically amenable to therapeutic intervention, with the goal of tissue preservation leading to improved clinical outcomes in the long term (2).

The existing paradigm of clinical care provision in moderate/severe TBI focuses on the “one-treatment fits all” approach, with consensus-based physiologic targets applied to all-comers, regardless of demographics and injury pattern (2). Not surprisingly, recent retrospective evaluation of these guideline-based therapeutic strategies has demonstrated little impact on morbidity and mortality over the last 25-years, despite adherence to such treatment targets (3). This is echoed by additional studies highlighting the individual heterogeneity of cerebral physiome responses in moderate/severe TBI, using high-fidelity advanced multi-modal cerebral physiologic monitoring (4–7). Such work has gone further to demonstrate that much of the cerebral physiologic insult burden seen in moderate/severe TBI is resistant to current therapeutic interventions, calling for more personalized and directed approaches (8–12). Furthermore, aside from the lack of efficacy of guideline-based approaches, the ability to prognosticate in this population has been limited as well, with current population-based standard models accounting for less than half of the variance seen in outcomes (1, 13, 14).

Based on the above, it is readily apparent that the future in moderate/severe TBI care provision calls for two main advances: (1) Precision prognostication and (2) Development of personalized therapeutics directed at secondary injury mechanisms driving morbidity and mortality. Precision prognostication of long-term outcomes would facilitate improved communication between healthcare professionals involved in care provision, and between the care team and families, hopefully minimizing the uncertainty of the future. Further, such prognostication can be applied to higher temporal physiology/cellular states over the course of the patient's acute and subacute hospital stay, facilitating prediction of upcoming events and hopefully early mitigation/prevention. Personalized therapeutics directed at patient-specific secondary injury mechanisms, targeting molecular pathways driving such physiology or cellular dysfunction, would reduce insult burden, alter tissue-fate, and hopefully improving morbidity and mortality for moderate/severe TBI patients.

Though precision prognostication and personalized therapeutics concepts are simply stated, in TBI the practical steps involved in developments within these two areas is complex. Both require integration of complex omics data sets (1), consisting of comprehensive cerebral physiome (4–6), advanced neuroimaging modalities (15–19), proteome (20–24), and genome/epigenome (25–28). Such omics data would be ideally sampled serially, and combined with detailed patient demographics, treatment information, and clinical outcomes. With proper integrative analyses, these complex data sets could inform precise prognostication, tailored to the individual patient. Similarly, these data sets could inform a “top-down” approach to the development of personalized precision therapeutic regimens in moderate/severe TBI care.

Over the past decade, there has been a rapid expansion of omics data reported in the moderate/severe TBI literature. The complexity of such data limits the ability of the bedside clinician to interpret and translate this information into improved prognostication or individualized management. As such, there is a need for advanced integrative neuroinformatics approaches, harnessing techniques in data science and machine learning/artificial intelligence, so that precision prognostication and personalized therapeutics development may become a reality (29–31). Within this review, we begin with an overview of the current state of omics data in moderate/severe TBI, highlighting physiome, advanced neuroimaging, proteome, and genome applications. Next, we outline the current limitation of such data sets in clinical care provision. Finally, we outline a potential path forward to achieve precision medicine in moderate/severe TBI, using advanced integrative neuroinformatics, drawing from recent advances in other neuropathologies.

## Current Status of OMICS Data in Moderate/Severe TBI

Within the subsections below, we will highlight some of the pertinent recent literature with relation to the various omics data categories in human moderate/severe TBI. We outline recent discoveries in the physiome, advanced neuroimaging, proteome, and genome research. These sections are not meant to be exhaustive collections of advances, so we direct interested readers to the references and related publications in the field for more details if additional information is desired. The figures providing examples of high-frequency cerebral physiology data were obtained from our existing, approved (University of Manitoba REB: H2017:181, H2017:188, H2020:118) and previously published database work in moderate/severe TBI (9, 10, 32–35).

### High-Frequency Multi-Modal Cerebral Physiome

**Figure 1:**
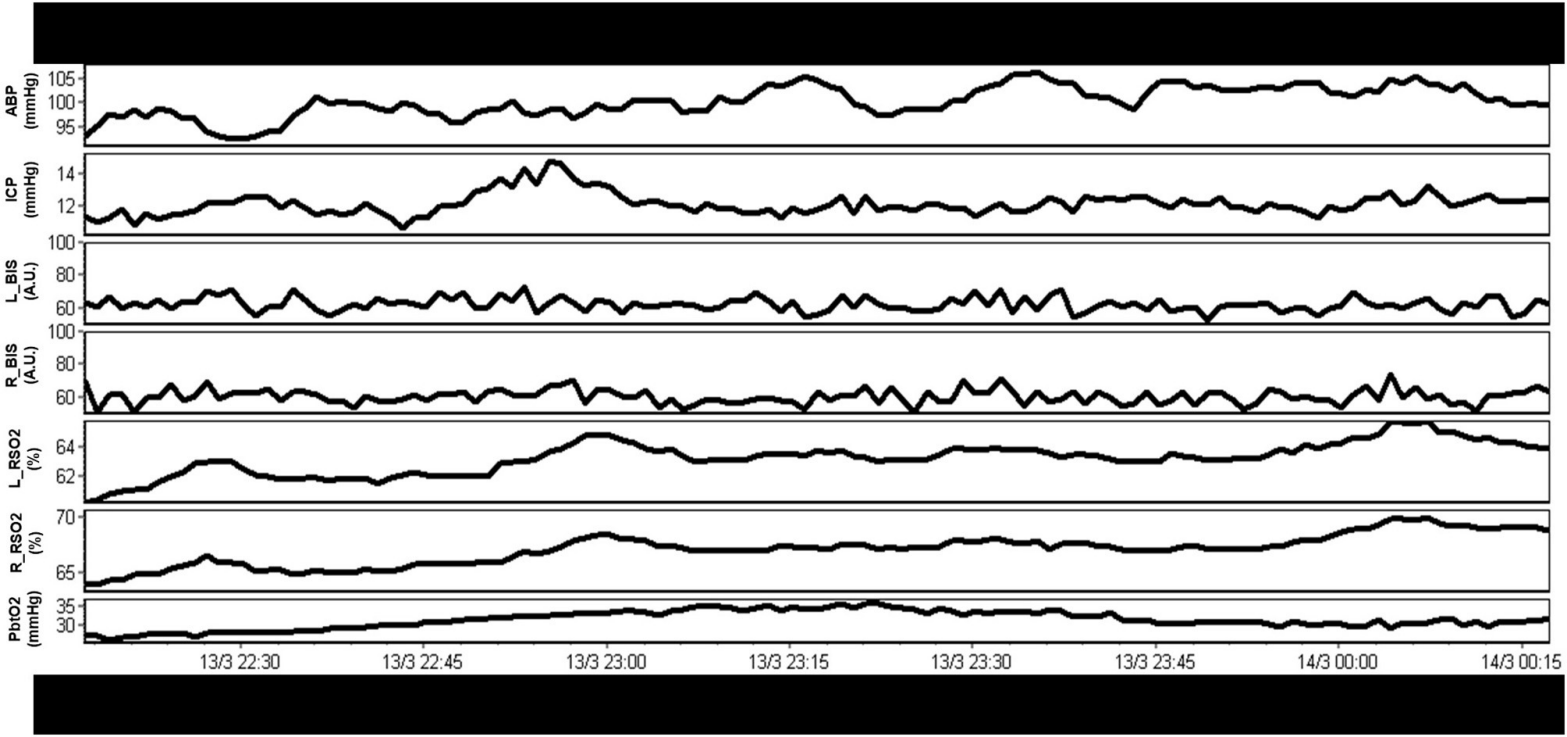
Example of raw continuous multi-modal cerebral physiologic monitoring in moderate/severe TBI. A rich variety of continuous physiologic parameters are currently monitored, however, interpretation of these raw parameters in concert is complex. A.U., arbitrary units; ABP, arterial blood pressure; BIS_L, bispectral index on left hemisphere; BIS_R, bispectral index on right hemisphere; ICP, intracranial pressure; mmHg, millimeters of Mercury; rSO_2__L, regional oxygen saturations on left frontal lobe (using near infrared spectroscopy); rSO_2__R, regional oxygen saturations on right frontal lobe (using near infrared spectroscopy); PbtO_2_, extracellular partial pressure of oxygen in the brain tissue; TBI, traumatic brain injury; %, percent. Data taken from previously published and approved studies (University of Manitoba REB: H2017:181, H2017:188 and H2020:118) (9, 10, 32–35).

Over recent decades there has been a dramatic expansion of continuous bedside multi-modal cerebral physiologic monitoring, beyond the classic intra-cranial pressure (ICP) and cerebral perfusion pressure (CPP) (4, 6). Such devices include continuous multi-channel near infrared spectroscopy (NIRS) based cerebral oximetry, (36) invasive parenchymal extracellular oxygen partial pressure monitoring (PbtO_2_) (6, 37, 38), invasive parenchymal thermal diffusion based cerebral blood flow (CBF) monitoring (39), transcranial Doppler (TCD) based cerebral blood flow velocity (CBFV) monitoring (40–42), and continuous electroencephalogram (EEG) (4, 43–45). Many of these devices output full-waveform level data, in high-frequency, with varying levels of literature supporting their association with patient's long-term outcomes (3, 4, 6, 46–49) and variable support from multi-disciplinary groups (4, 5, 50, 51). Some devices, such as PbtO_2_ monitoring, have shown such robust relationships between low PbtO_2_ and poor outcome, that randomized trials into ICP vs. ICP and PbtO_2_ directed therapies are now being undertaken (6). Thus, expanding the characterization of the physiome at the bedside improves our understanding and detection of secondary insult burden. Figure 1 provides an example of a continuous, raw, multi-modal cerebral monitoring data stream in moderate/severe TBI, and demonstrates both the variety and complexity of currently utilized modalities.

**Figure 2:**
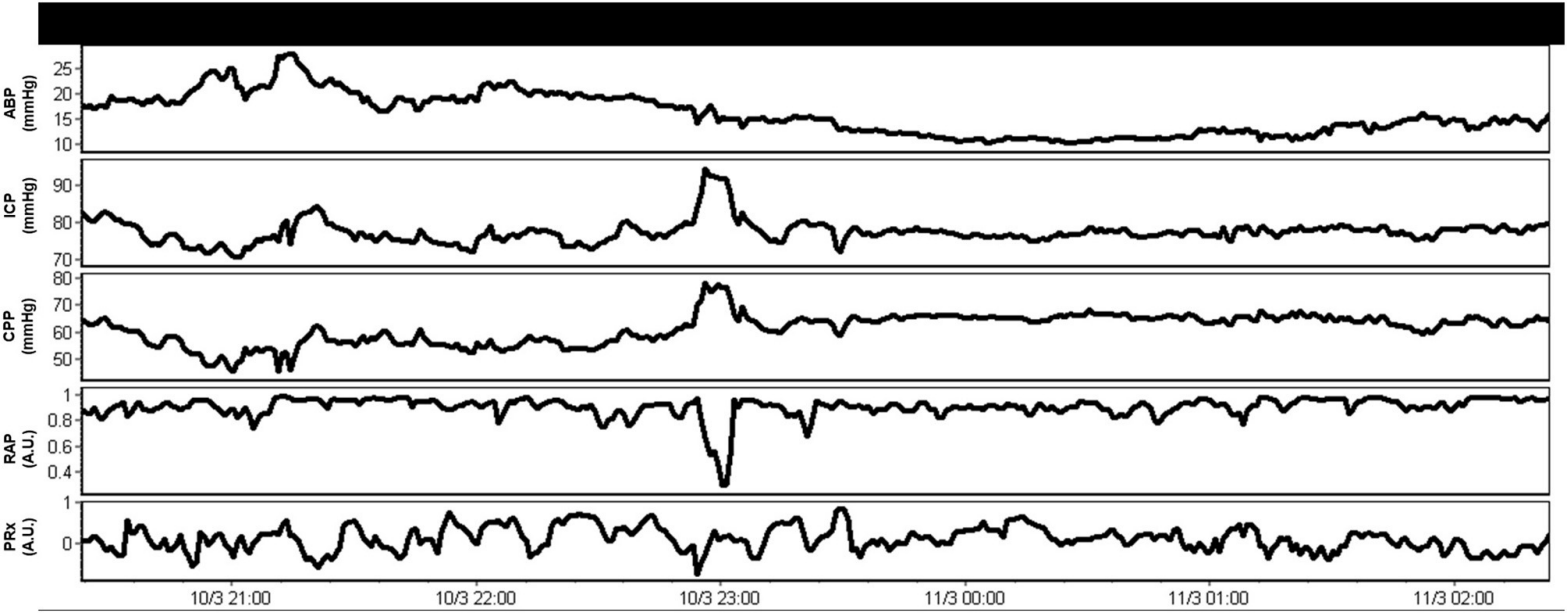
Example of combined raw and derived continuous multi-modal monitoring metrics in moderate/severe TBI. These derived continuous parameters can be computed in real time and made available to the treating clinician to help tailor management. A.U., arbitrary units; ABP, arterial blood pressure; CPP, cerebral perfusion pressure; ICP, intracranial pressure; mmHg, millimeters of Mercury; PRx, pressure reactivity index for cerebrovascular reactivity monitoring (moving correlation between ICP and ABP); RAP, compensatory reserve index (moving correlation between ICP and pulse amplitude of ICP). Data taken from previously published and approved studies (University of Manitoba REB: H2017:181, H2017:188 and H2020:118) (9, 10, 32–35).

**Figure 3:**
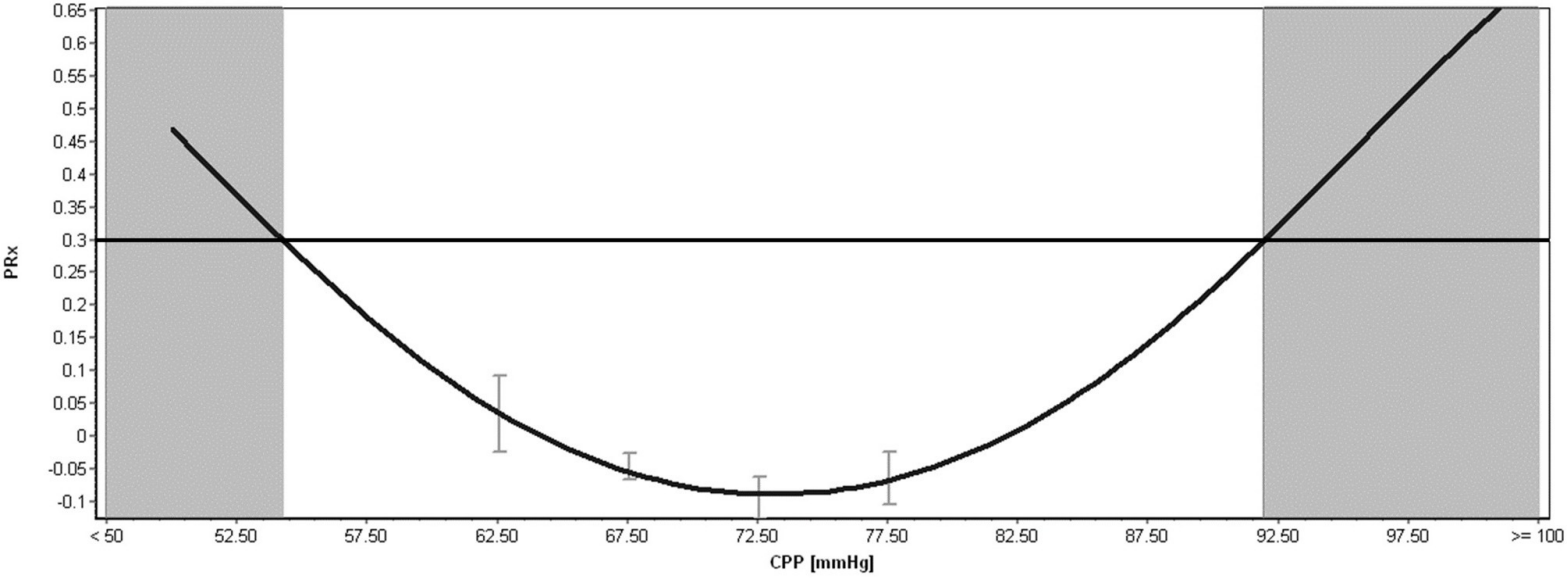
Example of Individualized Optimal CPP, Upper and Lower Limits of Regulation Calculation in Moderate/Severe TBI. CPP, cerebral perfusion pressure; mmHg, millimeters of Mercury; PRx, pressure reactivity index for cerebrovascular reactivity monitoring. The above plot shows an example of individualized optimal CPP estimation, calculated through error-bar plots of PRx vs. CPP and the fitting of a parabolic curve to the relationship. The minimum of this parabolic distribution represents the optimal CPP, based on the past 4-h of data. The shaded areas represent the lower limit of regulation (LLR; on the far left of the figure) and the upper limit of regulation (ULR; on the far right of the figure). The area in-between these two shaded zones represent a theoretical safe CPP range where autoregulation is mostly preserved (i.e., < +0.30), which in this example is a wide CPP range. Both the optimal CPP and LLR/ULR is continuously calculated over time using sliding windows of data, leading to continuously updating individualized bedside targets. Data taken from previously published and approved studies (University of Manitoba REB: H2017:181, H2017:188 and H2020:118) (9, 10, 32–35).

Aside from the raw physiologic parameters provided from these multi-modal devices, biomedical engineering principles applied to signal analysis has led to additional derived metrics of cerebral physiologic function. Such measures include continuous assessments of autonomic function (52), cerebrovascular reactivity/autoregulation (46, 47, 53), cerebral compensatory reserve (54, 55), and entropy indices (56–58). All such measures have demonstrated associations with patient outcome, many showing strong independent associations and an increased account of outcome variance beyond standard ICP/CPP monitoring, when adjusting for baseline patient characteristics (48, 49, 59). In addition, many of these measures remain independent to current guideline-based therapeutic interventions in moderate/severe TBI care (11, 12), highlighting the need for additional precision therapeutics aimed at the physiologic insult burden/dysfunction that such metrics are monitoring. This ever-growing literature body is further bolstered by the recent acceptance of these parameters by multi-disciplinary consensus groups as important adjunct monitoring variables for TBI care (4, 5, 51). Furthermore, in the spirit of transitioning to more personalized treatment regimens, there has also been recent work describing individualized physiologic targets/thresholds. Such personalized thresholds include continuously updating optimal CPP (CPPopt) (60–64), individualized ICP thresholds (65, 66), and individualized depth of sedation targeting (34), all based on cerebrovascular reactivity monitoring and its relationships with CPP, ICP, and EEG entropy index, respectively. These individualized physiologic targets have shown stronger associations with patient outcome, compared to current guideline “one target fits all” thresholds for CPP and ICP (66, 67). Figure 2 provides an example of continuous derived cerebral physiologic metrics in TBI and demonstrates their ability to be computed in real time, in concert with raw physiologic parameters, enabling them to guide bedside management. Figure 3 provides an example of personalized CPP targeting using cerebrovascular reactivity metrics.

However, despite the promising nature of multi-modal monitoring of the cerebral physiome, the sheer volume of data presented to the clinician often leads to information overload, clouding one's ability to make informed changes in therapeutic intervention for TBI patients. In attempts to improve understanding of such complex high-fidelity data sets, machine learning techniques have been applied (49, 68, 69). Such work has preliminarily led to improved understanding of the multi-variate relationships between multi-model data sets, aided in deriving prognostic models based on patient demographics and cerebral physiology, and set the stage for potential forecasting of cerebral physiologic events into the future using either invasive and non-invasive techniques. Yet, such efforts remain in their infancy, often requiring advanced data science and engineering skill sets for appropriate analysis. Further, integration with other omics data streams has been limited to only a few small biomarker (21, 70) and neuroimaging studies (71–73). As such, there is a desperate need for uniform and comprehensive integration of these complex physiome data sets with other omics data, to facilitate improved prognostication across different time scales, and highlight potential molecular pathways of cerebral physiologic dysfunction that are amenable to precision therapeutic intervention.

### Advanced Multi-Modal Neuroimaging Modalities

TBI can be detected using various imaging techniques such as plain head computed tomography (CT), CT angiography (CTA), CT perfusion (CTP), and magnetic resonance imaging (MRI), during the acute phase of care. In the subacute and long-term phases of care, correlation between imaging and clinical phenotype can involve an expanded set of advanced neuroimaging modalities, such as: positron emission tomography (PET), MRI perfusion imaging, functional MRI (fMRI), and diffusion tensor imaging (DTI) to study brain metabolism, CBF, as well as functional and structural connectivity, respectively. Below, we briefly touch on these main categories of imaging modalities in TBI. Table 1 provides a summary overview of selected imaging modalities in TBI.

**Table 1:** Categories of advanced neuroimaging in moderate/severe TBI.

**Clinical tools**	**Diagnoses**
Plain head CT	Plain head CT accurately and promptly diagnoses structural abnormalities such as skull fractures, intracranial bleeds, brain contusions, and brain herniation. But does not provide any functional information about the brain.
CT angiography	CTA provides anatomic information about the brain blood vessels such as injury or traumatic occlusion of the blood vessels.
CT perfusion	It is an advanced CT scan that provides both functional and anatomic information about the brain, and it is mainly used in triage patients with acute stroke. This imaging techniques quantifies perfusion parameters such as cerebral blood flow and cerebral blood volume.
Magnetic resonance perfusion	Magnetic resonance perfusion is non-invasive and can be used to detect intracranial arterial blood flow. It can also detect perfusion parameters of affected brain tissues such as cerebral blood flow and cerebral blood volume.
Diffusion Tensor Imaging	Diffusion tensor imaging (DTI) is a promising technique that can provide vital information about microstructural changes and structural connectivity (e.g., white matter tracts) within the brain.
Positron emission tomography	With right radiotracers, Positron Emission Tomography (PET) can estimate specific molecular abnormalities (e.g., phosphorylated tau and neuroinflammation) or brain glucose utilization with reasonable spatial resolution.
Functional magnetic resonance imaging	With advanced analytic techniques, intrinsic connectivity networks (ICNs) can be derived from fMRI data, the abnormality of which has been implicated in TBI and many other disorders of consciousness.

*CT, computed tomography; CTA, computed tomographic angiography; DTI, diffusion tensor imaging*.

#### Computed Tomographic Based Approaches

Plain head CT evaluates the anatomical changes in the brain structures and CTA evaluates anatomical changes in the brain blood vessels, secondary to the traumatic injury. While plain CT and CTA are a good quick assessment of the anatomical changes, these do not provide any functional information of the brain. Furthermore, many of the interpretations are qualitative, as opposed to quantitative, though some advances in quantifiable artificial intelligence based interpretation have emerged (16, 72). In contrast to plain CT and CTA, CTP is an advanced imaging technique that is useful in the detection of TBI and brain death as it provides both an anatomical and functional assessment of the brain (19). In most emergency departments CT is the predominant imaging modality for patients with TBI as it is fast, widely available, and has minimal contraindications. While technologic improvements have reduced the degree of radiation exposure, this still remains a notable disadvantage of CT imaging.

CTP at hospital admission has been proposed as a means to predict the in-hospital mortality in patients with severe traumatic brain injury. However, this needs to be further validated and is currently the subject of an ongoing larger prospective study (NCT04318665). If found to be accurate, CTP may become a valuable initial prognostic tool at the time of presentation that helps inform appropriate clinical management. This is exemplified through the principle of brain death declaration by CTP, where a marked decrease in the CBF and cerebral blood volume (CBV) in the brainstem has been shown to be the most sensitive and specific imaging ancillary test with the highest positive predictive values (74–77). This could help reduce resource intensive but futile care in those who are already brain dead at the time of hospital admission and may facilitate the precious gift of organ donation.

#### Magnetic Resonance Imaging Approaches

Although MRI can provide superior tissue contrast and spatial resolution compared to CT, MRI is not the preferred modality of imaging in the emergency room setting due to its relatively high cost, limited availability within or immediately adjacent to the ER, and slow image acquisition. Additionally, screening for the presence of contraindications is often not possible in those with moderate and severe TBI at the time of presentation. However, when available and when possible, MRI can provide valuable anatomical information, and methods such as MRI-based susceptibility weighted imaging (SWI) have been shown to be particularly sensitive for detecting micro-hemorrhages in TBI patients. However, these microbleeds are not necessarily colocalized with the microstructural changes (78). Nonetheless, while CT continues to be predominantly used for assessment of TBI patients in the ER at present, MRI techniques hold significant promise for the future.

#### Magnetic Resonance Perfusion Approaches

Consideration of the dynamics of CBF/CBV and perfusion can be facilitated through advanced magnetic resonance perfusion (MRP). This is non-invasive and can be used to obtain the functional information like that from CTP to detect CBF. However, for the reasons listed above, MRP has yet to be commonly used in the clinical setting for severe TBI patients (53).

#### Diffusion Tensor Imaging Approaches

Diffusion tensor imaging (DTI) is an advanced MRI technique that is based on the diffusion of protons in neuronal axons and provides vital information on the structural connectivity within the brain. The two main clinically relevant DTI parameters are fractional anisotropy (FA) and mean diffusivity (MD). FA measures the anisotropic diffusion of protons within a voxel and MD depicts the average diffusion of protons over all sampled directions. Normal white matter of the brain usually demonstrates anisotropic diffusion along the direction of the axons resulting in a high FA and low MD. However, in patients with TBI, traumatic axonal injury results in altered FA and MD measurements in the various involved brain regions to a degree dependent on the severity of the brain injury (79). In one study, of the regions examined, 88 and 92% had significantly lower FA in mild and moderate/severe TBI subgroups, respectively, compared to the controls. MD was found to be higher in 95 and 100% of the brain regions examined in those with mild and moderate/severe TBI, respectively, when compared to controls. A recent meta-analysis showed that high FA and low MD is associated with better cognitive outcomes (80). However, more research is needed before this important technique is used in routine clinical practice to inform individual patient decision making (81).

#### Positron Emission Tomography Approaches

The biggest advantage of PET over other *in vivo* imaging methods (e.g., MRI or CT) is the possibility to investigate specific biochemical and cellular processes by modifying the tracer used. Fluorodeoxyglucose (FDG), a radiolabeled glucose analog, is the most widely used PET radiotracer as it allows one to estimate regional glucose uptake, which is useful for delineating hypermetabolic tumors. In TBI, FDG uptake is known to be elevated immediately following trauma for a short period of time (up to 3 h), followed by a longer term decrease lasting days to weeks (82). A reduced degree of hypometabolism, in the sub-acute phase, has been associated with better long term outcomes (83, 84). However, precise mechanistic attribution of FDG PET observations to singular events has been elusive as FDG uptake can be affected by several processes, including inflammation (increase), cell dysfunction (increase or decrease), atrophy (decrease), and neurotransmitter activities (increase or decrease).

Other radiotracers of interest in TBI research include those that are sensitive to tau proteins and translocator proteins (TSPO). Among many, tau PET tracers (e.g., [^18^F]flortaucipir) has garnered the most interest as it enables quantification of phosphorylated tau *in vivo*, and thus directly addresses accumulation of the neuropathological hallmark of chronic traumatic encephalopathy (85, 86). The biggest limitation of tau PET imaging is its unspecific binding to beta-sheets (e.g., beta-amyloid plaques and alpha-synuclein) and other molecular structures (87). TSPO is a surrogate marker for microglial activation and astrogliosis; thus, TSPO PET yields a new opportunity to quantify neuroinflammation and has gained traction in the field (88). However similar to tau PET tracers, TSPO tracers have not demonstrated the desired specificity (89), again limiting definitive mechanistic inferences. Additionally, 2nd generation TSPO tracers have anrs6971 polymorphism-dependent binding affinity which further limits their generalizability (90).

#### Functional Magnetic Resonance Imaging Approaches

Although there are actually different types of fMRI contrast (91), blood oxygen level-dependent (BOLD) fMRI is by far the most common, and is based on the principle that elevated regional CBF (and there for increased oxygen delivery) follows neuronal activation in the brain in order to meet locally increased metabolic demand (92). The resulting spatiotemporal increase in oxygenated/deoxygenated hemoglobin ratio can be sensitively measured by acquiring a series of T2- or T2^*^-weighted MRI images, allowing signal changes over time to be interpreted as changes in local neural (neuronal and astrocytic) activity (92, 93). In 1997, Biswal and colleagues reported that BOLD fMRI signals also fluctuate synchronously in distant brain regions even when an individual is not engaged in a particular task (94). Those brain regions with synchronously fluctuating activities form intrinsic connectivity networks (ICNs) (95) ICNs are often characterized by independent component analysis (ICA) of resting-state low-frequency (typically ranging from ~0.01 to 0.1 Hz) fMRI data. Among different ICNs, the abnormalities of default mode network (DMN) have been frequently associated with TBI (96–99), and a very recent systematic review revealed that the most frequently studied MRI-based brain connectivity biomarkers for mild TBI to date are global functional connectivity and DMN functional connectivity (along with DTI measures of FA) (100).

The DMN is one of the most extensively studied brain networks, the disturbance of which has been associated with many neurological and psychiatric disorders that affects consciousness (101, 102). The DMN is situated in the posterior cingulate cortex (PCC) and medial prefrontal cortex (mPFC) with prominent nodes in the medial temporal lobe (MTL) and the angular gyrus (103). Following partial recovery of consciousness after an acute TBI that resulted in coma, patients in a minimally conscious or confused state showed partially preserved intra-DMN connectivity while such connectivity was absent in those that remained comatose (97). The DMN connectivity returned to a normal level upon full recovery of consciousness (97), although persistently altered DMN connectivity (>1 year since injury) predicted poor emotion recognition and social integration (96) as well as cognitive dysfunction (104) in TBI patients. While promising, more longitudinal studies with larger sample size are warranted prior to the wide adoption of fMRI for clinical use in TBI.

#### Machine Learning Approaches

The basic concept of most neuroimaging machine learning techniques is to classify patients based on human-annotated datasets. For example, Mitra et al. (105) trained a random forest classifier that identifies diffuse axonal injury-containing FA maps (DTI measurement) of TBI patients (*n* = 179) from healthy controls (*n* = 146) with moderate accuracy (~68%). Better accuracy was achieved when resting-state fMRI (84.1%) was used with support vector machine than when FA maps (75.5%) were used in different cohort of TBI patients (*n* = 50; control *n* = 50) (106). While these studies demonstrate a proof-of-concept of how machine learning approaches, using neuroimaging data, can be applied in TBI care, the true advantage of machine learning is achieved if the human-annotated labels are taken from follow-ups and linked to the baseline imaging data, which is used to train a prognostic classifier that can be used at an earlier stage (107, 108). To fully benefit from these recent technical advances, a large longitudinal neuroimaging database is necessary. This will allow for detailed phenotypic data to be linked to appropriately sized training, validation, and test sets, therefore generating the most robust neuroimaging based machine learning models.

### Serial Proteome of Serum, Cerebrospinal Fluid, and Extracellular Space

Protein biomarkers of tissue fate play a significant role in diagnostics, prognostics, and monitoring across a wide array of medical fields. In TBI, throughout the last decade, a number of proteins in serum have been suggested as having clinical utility. These include the primarily glial/astrocytic S100 calcium-binding protein B (S100B) and glial fibrillary acidic protein (GFAP), the neuronal Neuro-Specific Enolase (NSE) and Ubiquitin C-terminal Hydrolase L1 (UCH-L1) as well as the axonal Neurofilament-Light (NfL) and tau (109). By combining these markers with different cellular origins, as well as different serum kinetics (22), improved prognostication and assessment of injury severity can be achieved following moderate-to-severe TBI (110). S100B, which has been available in clinical assays for almost 20 years, has been implemented in guidelines in order to rule out the need for CT scanning in mild TBI (111). Further, it is used regionally to monitor unconscious TBI patients in order to detect lesion progression or development of neuronal insults during intensive care (112, 113). Other than S100B, only NSE exists in clinical assays [though GFAP was just approved in a point-of-care device (114)] and is used in guidelines for anoxic brain injuries following cardiac arrest (115). However, experimental clinical studies of longitudinal, serial sampling show that the other proteins exhibit similar capacity as S100B in detecting secondary neuronal insults in unconscious TBI patients (22). Additionally, S100B levels have been associated with the strength of resting-state brain connectivity in multiple resting-state networks on fMRI following severe TBI, highlighting its capacity as a global brain injury marker (116).

In terms of multiplexing the proteome following TBI, these studies are scarcer, and almost all have focused on profiling the proteome of either the CSF (117–120) or brain tissue (121) following injury. In a study of three TBI patients by Hanrieder et al. serial sampling of CSF, in the first 2 weeks following injury, using a shotgun proteomic approach showed that acute phase reactants, fibrinogens, as well as brain enriched proteins like GFAP and NSE demonstrated interesting temporal trends as tentative biomarkers (118). Proteomic approaches have been attempted by analyzing the brain extra-cellular fluid (ECF) cytokine response using multiplex techniques (24, 122–125), revealing interesting trends for some inflammatory proteins. Altogether, the field of proteomic profiling of brain fluids following TBI is still in its infancy as these mass spectrometry-based approaches are difficult to conduct on a larger scale, but new techniques in preliminary reports of both CSF and brain-ECF from TBI patients reveal several tentative markers of brain injury that may have clinical utility in the management of these patients.

Similarly, blood brain barrier (BBB) integrity and function may also be surveyed utilizing advanced proteomic approaches in TBI. Lindblad et al. demonstrated that the brain to blood clearance of S100B was associated with BBB integrity, while that for NSE was not (126), hence suggesting that different proteins might be more associated with BBB disruption than others. In another recent work by Lindblad et al. utilizing a protein-array targeting brain enriched and inflammatory proteins in 186 TBI patients, the extent of BBB damage was associated with increased complement proteins in CSF, presumably indicating an association between ongoing secondary injury, BBB disruption and a subsequent neuroinflammatory response (127). A similar trend could be seen in blood, but was more evident in the CSF compartment, highlighting the need for appropriate compartmental monitoring in order to accurately monitor pathology. Several inflammatory and structural proteins provided independent information in outcome prediction models. Thus, novel proteomic approaches reveal that BBB-disruption is a key event following TBI and seems to be associated with neuroinflammation, both clinically relevant secondary injuries that should be acknowledged for future research.

Finally, metabolic failure following TBI has been associated with different pathophysiological conditions, however the most detrimental is considered to be mitochondrial dysfunction (128). This is a condition that has been identified following TBI in animal models (129, 130), where different techniques exist to detect mitochondrial dysfunction. However, many of these laboratory techniques are not applicable *in vivo* in humans (131). Instead, in order to assess biochemical substances in the ECF *in vivo*, cerebral microdialysis (CMD) is commonly used (70). CMD consists of a probe of a semi-permeable membrane where a carrier fluid is being pumped through it, and in the process, extracting ECF substances through diffusion. This allows for serial sampling and thus consecutive bedside monitoring of focal cerebral metabolism. There have been several consensus meetings dealing with how to best optimize CMD as a clinical tool for metabolism monitoring, the most recent published in 2015 (132). The primary markers of metabolism that are monitored clinically in brain ECF are glucose, lactate and pyruvate.

In the case of a deranged cerebral metabolic redox state because of impaired mitochondria, there will be an accumulation of ECF brain lactate (as a surrogate marker for NADH) and a subsequent decrease of ECF pyruvate levels (a marker of NAD^+^) (133). An ECF lactate/pyruvate ratio (LPR) >25 has been independently associated with an unfavorable outcome following TBI (21). A deranged LPR retrieved from CMD has also been associated with an impaired phosphocreatine/ATP ratio, as a sign of an effect on the brain energy state (134). The association of brain ECF LPR and mitochondrial dysfunction is also supported in large animal experiments where cyanide is administrated, resulting in a prompt LPR increase (135). However, in CSF, while being suggested as a marker of mitochondrial disease (136), LPR has not been seen to be a similar marker of deranged metabolism following TBI. Studies either do not show any difference between TBI and healthy controls (137), or fail to see an association with outcome (138), stressing the necessity for compartmental monitoring in order to tailor treatment strategies following TBI (139).

### Candidate Gene Studies and Genome-Wide Association Work

The role of genetic variations, such as single nucleotide genetic polymorphisms (SNPs), in the heterogeneity of outcomes following TBI has been known for some time and has been the focus of a recent review by Gomez and colleagues. Polymorphisms in candidate genes involved in neural repair, BBB integrity, neurotransmission, inflammation, as well as those associated with neurodegenerative disease have all been examined for their association with global, cognitive, and physiologic outcomes following injury (27). Table 2 provides a general overview of major categories of candidate SNPs that have been explored in TBI to date. We refer the interested reader to comprehensive reviews on the topic in TBI for more information if desired (25–28).

**Table 2:** SNP categories from candidate genes explored in TBI to date.

**Gene function**	Example genes*	**Possible polymorphism outcome associations**
Neurodegeneration	*APOE*	• Strong evidence for association with Global outcomes. • Equivocal association with neurocognitive outcomes.
Neuronal repair	*BDNF*	• Evidence for association with global outcomes mediated by age and BDNF levels. • Moderate evidence of association with neurocognitive outcomes.
Blood-brain barrier	*ABCB1, ACBG2, ABCC8*	• Equivocal evidence of association with global or neurocognitive outcomes. • Association with cerebral edema and ICP in acute phase.
Neurotransmitter metabolism	*COMT*	• Equivocal evidence of association with global or neurocognitive outcomes.
Inflammation	*IL-1α, IL-1β, IL-6*	• Equivocal evidence of association with global or neurocognitive outcomes. • Association with risk of post-traumatic seizures/epilepsy.

*ABC, ATP binding cassette; APOE, apolipoprotein epsilon; BDNF, brain derived neurotrophic factor; COMT, catechol-O-methyltransferase; IL, interleukin; SNP, single nucleotide polymorphism*.

* *Above SNP categories are not exhaustive, but merely represent general categories with examples of SNP's investigated in TBI to date*.

The *APOE* gene has been the subject of numerous outcome association studies due to its role in neurodegenerative diseases such as Alzheimer's disease. Three polymorphisms of the gene are commonly found, ε2, ε3, and ε4, of which the ε4 allele has been found to be associated with increased risk of late onset Alzheimer's disease (140). This led to its examination by Teasdale et al. in 1997, who found that those with an APOE ε4 allele were significantly more likely to have poor global outcomes at 6 months post-injury as measured by Glasgow Outcome Scale (GOS) (141). Since this seminal work, numerous studies, and meta-analyses have found the APOE ε4 allele to be associated with worse global outcomes following TBI (28, 142, 143). The ε4 allele has also been linked to worse cognitive outcomes following TBI with some studies finding the allele associated with worse memory, processing speed, and overall increased cognitive impairment (144–146). However, not all studies have found an association with worse cognitive outcome and so further work is needed (147–149). Currently, the association of the APOE ε4 allele with poor outcomes is thought to be mediated by abnormal lysing of its protein product resulting in neurotoxic byproducts that worsen secondary injury and impair recovery (28).

Genes involved in neuronal repair and survival have also been examined for their association with outcomes following TBI. The *BDNF* gene, which encodes brain-derived neurotropic factor (BDNF), has been examined for association between its polymorphisms and global and cognitive outcomes. Early work by Failla et al. point to an association with certain SNPs and global outcomes mediated by interactions with age and serum BDNF levels (150, 151). When examining associations with cognitive outcomes following injury, the rs6265 allele was found to be linked to improved recovery of executive function, working memory, processing speed and other cognitive domains when compared to wild-type alleles (152–154). However, conflicting results have also been reported with the variant allele being associated with worse cognitive outcomes (155, 156). The mechanism by which this may be mediated is poorly understood.

Association studies have also been conducted on genes encoding ATP-binding cassette (ABC) transporter proteins, which are integral in the function of the BBB. SNPs of the genes encoding some ABC proteins have been found to be associated with global and cognitive outcomes while others have not (157–159). Notably, polymorphisms in the gene encoding ABCC8, a member of the ABC transport protein family, have been associated with elevated ICP and CT findings of cerebral edema in the acute phase of TBI (158, 160).

Similarly, polymorphisms in the genes that encode proteins involved in neurotransmitter metabolism, such as catechol-O-methyltransferase (COMT), have yielded contradictory results (160, 161). Association studies examining polymorphisms in genes integral to inflammation have also failed to provide uniform results (162–164). However, one study did find that polymorphisms in the gene encoding the inflammation associated protein IL-1β carried different risks for post-traumatic seizures/epilepsy (165).

Conflicting results and replication issues in candidate gene studies are well-documented in the field of human genetics (166) and reflect the need for unbiased genome-wide association studies (GWAS) to be performed in large, comprehensively phenotyped TBI cohorts. GWAS findings for complex traits have been shown to be more reliably replicated and are able to give insights into novel biology (167). For example, they can identify novel drug targets, infer the most relevant cell types and define the genetic heritability of the traits being studied. Future studies should genotype TBI cohorts to assess the predictive capabilities of polygenic scores derived large-scale GWAS from TBI-related traits in stratifying clinical outcomes in a standardized manner (168). Large TBI cohorts would also allow for the identification of genome-wide significant loci that have the potential to be novel therapeutic targets (169). Current initiatives that can facilitate GWAS in TBI include the Genetic Associations in Neurotrauma (GAIN) Consortium, the Enhancing Neuroimaging Genetics through Meta-Analysis (ENIGMA) Consortium, and the TBI-related focus of the Psychiatric Genomics Consortium-PTSD Working Group (PGC-PTSD) (17, 170) However, more focus needs to be placed on efforts to recruit and genotype research participants that have experienced moderate/severe cases of TBI. Further, significant work needs to be done before the evaluation of target gene polymorphisms are integrated into TBI care. However, a better understanding of the contribution of genotypic variation in global and cognitive outcomes following injury may aid in prognostication and eventually may allow for more personalized therapeutic targets.

## Limitations in the Application of Current Comprehensive OMICS Data in TBI

The main limitation associated with omics data streams in TBI is the overall complexities inherent with each data type. In general, integration of each omics data type is difficult given different temporal and spatial resolutions, requiring multi-disciplinary expert collaboratives in order to achieve such goals. This is also the case when adding baseline patient characteristics, treatment information, and serially assessed comprehensive outcomes. Aside from this, each data stream has its own specific considerations, which have limited their integration for precision medicine approaches in moderate/severe TBI.

Multi-modal high frequency physiologic data sets carry a few limitations. Data is typically streamed in full physiologic waveform format, leading to high data storage requirements, specialized data management, and biomedical engineering skills for processing and analysis. This is particularly the case for some of the more advanced derived metrics of cerebrovascular function, compensatory reserve assessments, and individualized CPP and ICP target characterization (34, 60–62, 65, 66). Further, with expansion of the various continuous cerebral physiologic monitoring possibilities, there now exists substantial volume of concurrent continuous data streams presented to the treating clinician, leading to information overload, and difficulties in knowing which information to act on. Finally, many of these physiologic data streams are only available during the acute phase of care, given their invasive nature. Thus, serial follow-up assessments of continuous cerebral physiology have classically not occurred. Though this has recently changed with advances in non-invasive monitoring and biomedical engineering techniques (171–174).

Advanced neuroimaging platforms suffer from a lack of temporal resolution, sacrificed for the sake of spatial resolution. As such, these snapshots of cerebral structure and connectome, have limited translatability to bedside care and integration with more continuous omics data streams. This is especially the case in the setting of critically ill moderate/severe TBI patients, where transport and care provision during lengthy advanced neuroimaging sequences are often not feasible, except for a few centers globally with advanced imaging platforms and critical care units co-located. Consequently, the costs associated with facility development, maintenance, need for specialized personnel (such as radiochemists for advance PET tracers, or MRI physicists for novel sequence development) and image acquisition at the individual study level, can be prohibitive for many centers globally.

Proteome biomarker data suffers from slightly different complexities. The lower sampling frequency of such serially measured samples makes it difficult to integrate with higher resolution physiologic data streams that typically drive bedside decision making in the acute phase of moderate/severe TBI (20, 22, 70). Though sampling frequency typically outstrips that of neuroimaging. Furthermore, given the above limitation of follow-up continuous physiologic assessments, many long-term serial biomarker studies have failed to be linked to cerebral physiology, limiting interpretability. Finally, most work to date has focused on small sets of protein biomarkers of interest, such as pro-inflammatory cytokine profiles (23, 123, 125), given the need for expertise in sample management, processing, and interpretation biologic significance. As such, proteome-wide analysis has not been conducted and may shed further light on molecular pathways driving secondary injury in TBI.

Finally, genomic and epigenomic data suffers two main issues in the TBI population. Sample size in TBI research has classically been quite small, in comparison to other genomic studies in non-neurological populations. Most moderate/severe TBI studies have <1,000 patients (26, 28). Thus, genome-wide approaches are left as exploratory initiatives, with limited conclusions drawn at the population level. Further to this, the volume of information obtained through current sequencing platforms is extensive and has limited investigations in this area to a few specialized centers. Integrating such data sets with other serially sampled omics data streams is also a challenge, particularly when attempting to comment on potential molecular pathways driving secondary injury.

## Integrative Neuroinformatics: Future of Precision Medicine in TBI

Based on the above-mentioned complex data streams, and limitations associated with their application, there exists a clear need for the use of advanced data science strategies to facilitate better understanding. As mentioned, some preliminary application of machine learning techniques has been conducted in high-frequency multi-modal cerebral physiologic data, development of automated neuroimaging-based predictions, and prognostic model derivation in moderate/severe TBI. However, such advances have yet to translate to wide sweeping changes in bedside care, or personalized precision medicine. Given the intrinsic complexity of TBI and the massive amount of information “encrypted” in most data modalities, in practice, there are multiple challenges for patient screening and for obtaining a better explanation of the individual neuropathological mechanisms. That is, collecting the above-mentioned data streams is a necessary but not sufficient condition for achieving a better understanding of the underlying neuropathological mechanisms and preventing clinical deterioration. In practice, comprehensive integrative neuroinformatics platforms are required to drive precision prognostication and derivation of personalized therapeutics targeting secondary injury mechanisms. As such approaches would be novel in moderate/severe TBI, here we propose a brief roadmap for the adaptation of significant advances recently made in other neuropathological conditions, such as neurodegenerative processes.

First, predicting the individual course of a progressive neurological condition, such as moderate/severe TBI, is of crucial importance for early diagnosis and optimum treatment selection. The last decade has seen a tremendous advance in neuroinformatic methods aiming to track disease advance in the context of most-prevalent neurodegenerative disorders (175–178). Sophisticated mathematical and statistical models, including novel Artificial Intelligence (AI) techniques, are increasingly allowing to decode the “encrypted” patterns in big neuroscience data. In general, neuroinformatic models of disease progression (DP) combining multi-modal data (electrophysiological, peripheral, genetics, imaging, clinical, etc.) with advanced computational techniques can be classified in two main categories: empirical and mechanistic. Empirical DP models (177) focus on making disease predictions (e.g., *which* subjects will develop the disease vs. *which* subjects will not) but without offering a detailed biological explanation of the underlying neuropathological causes. On the other hand, mechanistic DP models (29, 179–181) may make predictions, but their primary goal is to clarify disease mechanisms (e.g., *which* genes or brain areas are driving the disease, and *why*).

**Figure 4:**
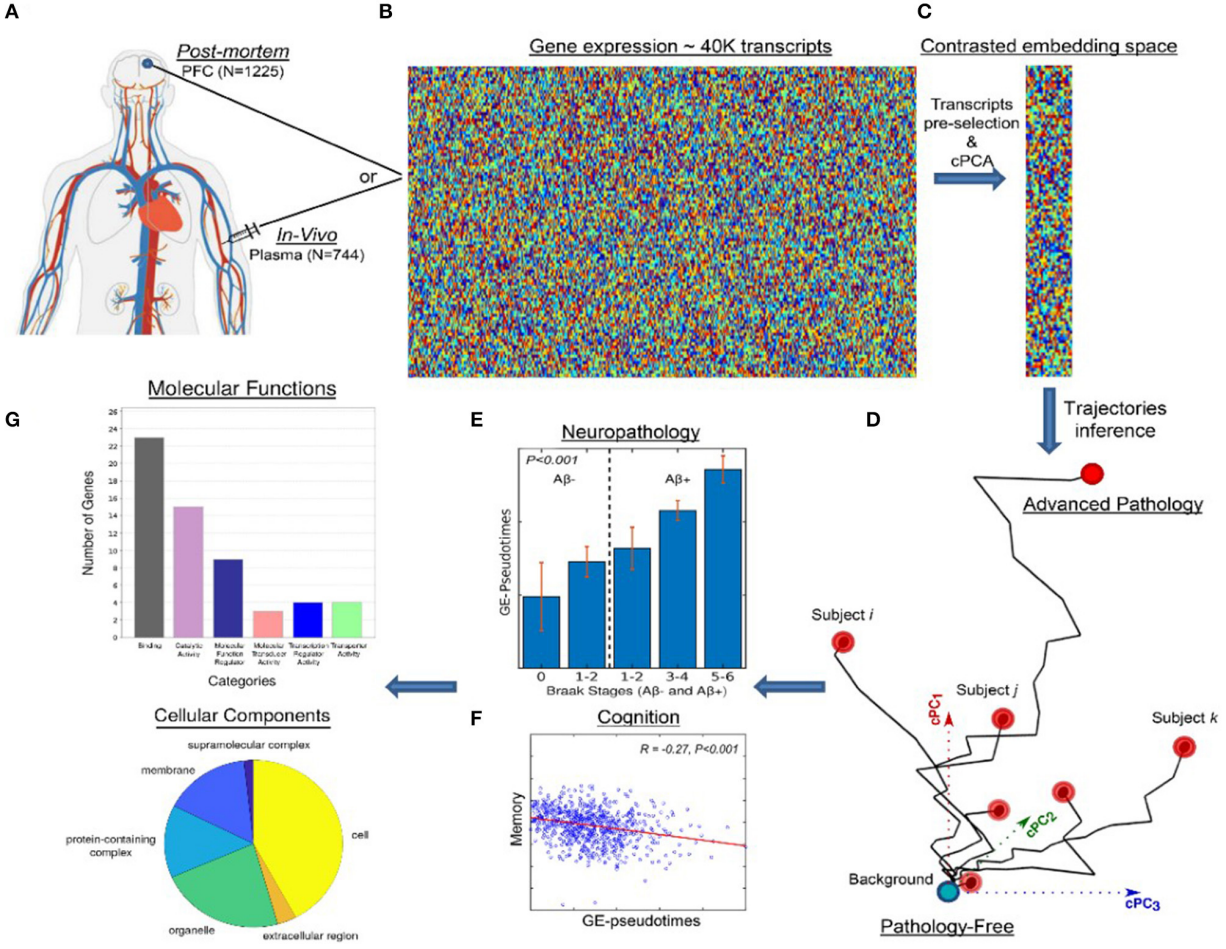
Schematic approach for detecting molecular disease-associated patterns and patient staging. *In-vivo* blood (*N* = 744; ADNI) and post-mortem brain (*N* = 1,225; ROSMAP, HBTRC) tissues **(A)** are screened to measure the activity of ~40,000 transcripts **(B)**. This high dimensional data is reduced to a set of disease-associated components **(C)** via contrastive PCA and/or variational autoencoders 0.171. This allows to represent each subject in a disease-associated space **(D)** where the corresponding position reflects her/his pathological state (with proximity to the left bottom corner implying a pathology-free state and to the right top corner implying advanced pathology). An individual molecular disease score is then calculated, reflecting how advanced is each subject in his/her disease-trajectory. This score significantly predicts neuropathological **(E)** and cognitive measurements **(F)**. Finally, the resulting model weights allow the direct identification and posterior functional analysis of most influential genes **(G)**. Image adapted with permission from Iturria-Medina et al. (183).

In the moderate/severe TBI context, it is critical to consider the distinction between these two DP modeling categories, while empirical approaches can represent powerful predictive tools, they constitute “black-boxes.” This handicap is not common to mechanistic models, which (in part to remain interpretable) often achieve a lower predictive capacity. In TBI applications, in order to maximize the tradeoff between the models' clinical predictability and biological interpretability, it will be essential to combine the advantages of empirical and mechanistic brain disease models (182). For example, in a recent neurodegeneration study (183), state-of-the-art machine learning advances for exploring and visualizing high dimensional data (184) were used to define contrasted disease trajectories and clinically screen the patients. This semi-unsupervised method (named *contrasted Trajectories Inference* [cTI]) uncovers the underlying neurodegenerative path in large-scale omics data, accurately identifying the series of sequential molecular states (genetic alterations) covering decades of disease progression, and subsequently revealing the relative position of each individual subject in that path. When applied to *in-vivo* microarray gene expression data from the blood of 744 subjects in the late-onset Alzheimer's disease (LOAD) spectrum (ADNI data; Figures 4A,B), cTI automatically identifies disease-associated patterns of genes that mirror neuropathological and clinical alterations (Figure 4C), and subsequently detects the relative ordering of individuals that better align with those patterns (Figure 4D). That is, by finding the relative position of each subject on the long-term disease “timeline” (Figure 4D), cTI provides a personalized molecular disease index that significantly predicts individual neuropathology (tau, amyloid and infarct positivity), cognitive deterioration and future clinical conversion (Figures 4E,F). Similarly, when evaluated on 1225 *post-mortem* brains in the spectrum of AD and Huntington's disease (ROSMAP and Harvard Brain Tissue Resource Center data [HBTRC]), it strongly predicts neuropathological severity and comorbidity (Braak, Amyloid, and Vonsattel stages). A notable attribute of the cTI approach is that it allows estimating the specific contribution of each gene on the identified disease “timeline” and the obtained personalized molecular disease index, based on the analysis of the model's resulting weights/loadings for each biomarker included in the original data (i.e., ~40,000 gene transcripts). Simply put, this predictive and semi-mechanistic technique overcomes the traditional AI “black-box” limitation, allowing the direct discovery of genes and related molecular pathways underlying disease evolution.

**Figure 5:**
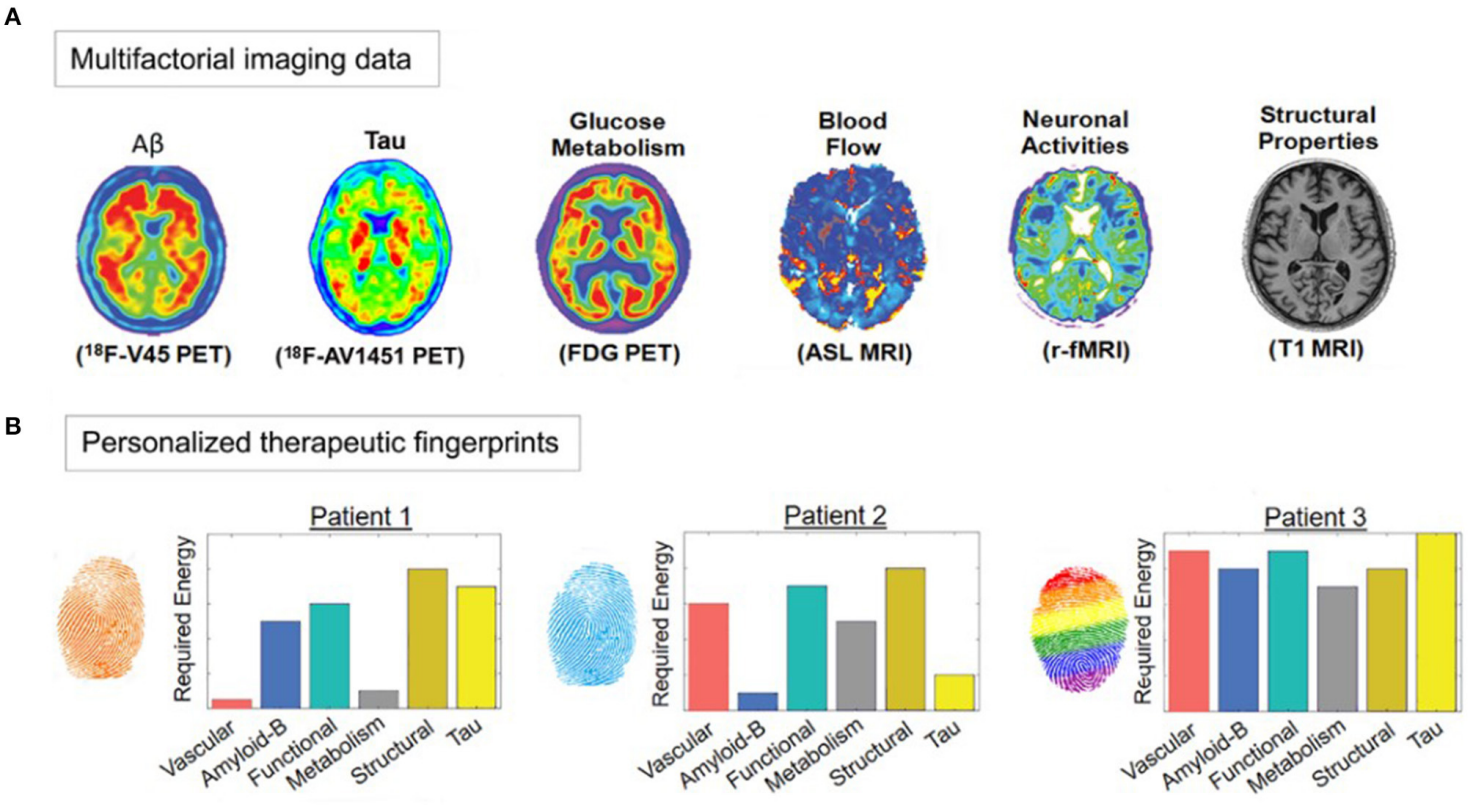
Imaging-based therapeutic intervention fingerprinting approach **(A)** Amyloid, tau, and F-fluorodeoxyglucose (FDG) PET, and Cerebral Blood Flow (CBF), functional activity at rest and gray matter density from MRI. A network-based approach allows individual characterization of the intra-brain direct factor-factor biological interactions and the spreading mechanisms through vascular/anatomical connections. Inverting the model's equations allow estimation of the optimum required to produce a desired clinical state (e.g., healthy). **(B)** Different pTIF patterns for three participants with similar clinical status. Note that Patient 1 requires lower cost-energy values for vascular and metabolic interventions, while Patient 2 requires lower values for anti-Aß and anti-tau interventions, suggesting the identification of specific single-target therapies that may benefit these patients (e.g., physical exercise and aducanumab, respectively). However, Patient 3 requires high cost-energy for all the single-target interventions, suggesting that combinatorial (and not single-target) treatments will be most beneficial. Image adapted with permission from Iturria-Medina et al. (185).

Computationally, it is also feasible to go in the inverse methodological direction (from characterizing mechanisms to performing treatment-effect predictions). In another recent neurodegeneration study by the same group, a neuroimaging-based personalized Therapeutic Intervention Fingerprint (pTIF; Figure 5) was introduced, aiming to characterize brain mechanisms for subsequently predicting individual treatment needs (185). Based on spatiotemporal analysis of multi-modal imaging data (i.e., PET, MRI, etc.), the pTIF demonstrates that the patients may need different treatments, not only depending on their brain's unifactorial alterations (e.g., tau and amyloid accumulation or not, dopamine alteration or not, atrophy or not) but also on their individual multifactorial brain dynamics: how the different biological factors interact and how they would respond (at the individual level) to potential clinical perturbations. In summary, after quantitatively characterizing basic disease mechanisms at the individual level (e.g., intra-brain spreading of tau and amyloid proteins, and their toxic interaction with vascular and structural factors), the pTIF integrates large amounts of data (e.g., millions of multi-modal brain imaging measurements) into a simplified individual patient profile of the quantitative biological factor modifications needed to control disease evolution. Results in aging and LOAD (ADNI data, *N* = 331; see Figure 5) support that pTIF allows to categorize the patients into distinctive therapy-based subtypes. These subtypes were relevant by comparing them to the patients' individual genetic profiles, finding that each pTIF-subtype presents a distinctive/characteristic pattern of gene expression alterations in the blood.

In summary, to achieve true personalized care in moderate/severe TBI, it is vital to use advanced neuroinformatic tools. For this, the combination of both empirical (highly predictive) and mechanistic (biologically interpretable) disease progression models is an indispensable associated step, as important as the acquisition of multifactorial data (molecular, electrophysiologic, neuroimaging, clinical, etc). Deep mathematical and computational modeling of multidimensional neurological disorders is, however, complex and time demanding. The TBI research and clinical community could take advantage of the growing number of techniques initially developed for other neurological conditions (Alzheimer's, Parkinson's, depression), which have been tested and validated in large-scale heterogenous datasets. As such, the use of already available Open-Access neuroinformatic tools (177, 178, 182, 186), integrating a large variety of biological data for a better understanding of individual disease progression and treatment needs, may accelerate the adaptation and improvement of advance computational approaches in the moderate/severe TBI context.

## Leveraging Integrative Neuroinformatics Discoveries for Precision Medicine

Integrating comprehensive serial physiome, neuroimaging and proteome information, with genome/epigenome data from multicenter clinical data sets, will facilitate highlighting various pathways of cerebral physiologic dysfunction of interest which are driving current poor outcomes. Using this information in a top-down fashion, cellular/small animal and large animal models could then be developed to explore these in more detail. Here, detailed probing of molecular drivers of secondary injury could occur, with comprehensive pathway elucidation and characterization of connectomic and histopathological consequences of dysfunction. Understanding here would be expected to lead to discovery of precision targets aimed at prevention and intervention for such secondary injury mechanisms. Precision therapeutics would then need to be further explored in controlled healthy and TBI small animal models, to gain understanding of connectomic, physiologic, and tissue consequences of such interventions. However, in order to circumvent past failure of clinical translation from cellular/small animal models, large animal TBI models would need to be employed as well.

Cellular/small animal and large animal platforms would be complementary and poised to inform one another in the process of target elucidation and precision therapeutics development. Comprehensive multi-modal high-resolution cerebral physiologic monitoring would be applied in large animal models, with serial serum and extracellular fluid sampling for protein biomarkers and whole brain explantation post-experiment for evaluation of histopathological correlates. Such platforms would facilitate directed validation of emerging personalized medicine approaches to TBI care, exploration of precision therapeutics directed at secondary injury pathways, and assessment of histopathological consequences of various interventions in detail on a model which closely represents the human cerebrum in structure and physiology. Such large animal platforms would focus on moderate/severe TBI simulation and therapeutics development, to bridge the existing gap in translating small animal model discoveries to humans. Further, this platform would serve as the ultimate pre-clinical means of precision therapeutics investigation prior to implementation in humans. Discoveries here would lead to translatable changes to bedside care, focused on individualized care plans, and transitioning away from failed guideline approaches toward personalized medicine in TBI care.

## Fundamental Requirements for Future Work

Based on the integrative neuroinformatics framework presented here, there are some basic requirements to facilitate this in moderate/severe TBI. First, the comprehensive clinical phenotyping requires multi-center collaborations, in order to build enough power to adequately inform such prognostic models and top-down exploration of molecular pathways of secondary injury. This requires that each site has the capacity for serial high-resolution data capture for the physiome, proteome and neuroimaging. Similarly, it requires the basic infrastructure and personnel support to ensure complete data acquisition and proper bio sample storage. With the aid of existing specialized TBI research groups, such as the Canadian Traumatic Brain Injury Research Consortium (CRTC) and the CAnadian High-Resolution TBI (CAHR-TBI) research collaborative (33), such comprehensive data collection strategies are feasible. Second, multi-disciplinary groups of specialist clinicians and scientists are required. Expertise in epidemiology, data science, genetics, proteomics, advanced neuroimaging, biomedical engineering, cerebral physiology, and neuroscience are required to facilitate success with such integrative neuroinformatics approaches. Third, comprehensive pre-clinical platforms are necessary to facilitate exploration of molecular pathways of secondary injury. It is through cellular, small and large animal models of neural injury, that pathways uncovered by neuroinformatic approaches applied to comprehensive clinical data, can be explored in more detail. Cellular and small animal platforms would facilitate exploration of such molecular pathways, derive therapeutic targets, and highlight histopathological correlates. Similarly, given issues with translatability of findings from cellular/small animal TBI platforms in the past, large animal models of TBI are also needed to allow for further pre-clinical investigation of therapeutic targets, interventions and histopathological outcomes on cerebral structures more closely related to humans (187). Such large models are key in bridging the current bench-to-bedside gap in TBI research. Fifth, based on discoveries from the top-down approach to precision therapeutic development, translation to bedside care and trials is necessary. Study of such personalized approaches will differ from standard clinical trials, as intervention for each patient will be based on their individual phenotypic signature identified from presenting and early-phase omics data. Finally, clinical end-users are critical to involve in the entire top-down process, as their real-world bedside experience in caring for such complex patients can provide crucial insight into what may or may not be feasible for personalized precision medicine in this population.

## Conclusions

Current therapeutic treatment paradigms in moderate/severe TBI have failed to lead to substantial impacts on morbidity or mortality in the past three decades. Similarly, the ability to prognosticate in this population currently accounts for limited variance seen in outcomes. Recent expansions in phenotypic characterization of this population using current omics approaches have led to a rapid expansion to the data available to treating clinicians. The future of moderate/severe TBI care necessitates precision prognostication and therapeutics, tailored to the individual patient based on comprehensive physiome, advanced neuroimaging, proteome, and genome/epigenome. Such work will rely on integrative neuroinformatics platforms for precision prognostication and a “top-down” development of personalized therapeutics directed as secondary injury mechanisms driving morbidity and mortality.

## Author Contributions

FZ, YI-M, and CA: responsible for concept, design, and manuscript preparation. ET, JS, JK, CF, and GW: undertook review and screening of the literature as well as aided in preparing the manuscript. AG: undertook review and screening of the literature as well as aided in preparing the manuscript and figures. All authors contributed to the article and approved the submitted version.

## Funding

FZ receives research support from the Manitoba Public Insurance (MPI) Neuroscience/TBI Research Endowment, the Health Sciences Centre Foundation Winnipeg, the United States National Institutes of Health (NIH) through the National Institute of Neurological Disorders and Stroke (NINDS)(Grant #: R03NS114335-01), the Canadian Institutes of Health Research (CIHR)(Grant #: 432061), the Canada Foundation for Innovation (CFI)(Project #: 38583), Research Manitoba (Grant #: 3906), the University of Manitoba VPRI Research Investment Fund (RIF), the University of Manitoba Centre on Aging, and the University of Manitoba Rudy Falk Clinician-Scientist Professorship.

## Conflict of Interest

The authors declare that the research was conducted in the absence of any commercial or financial relationships that could be construed as a potential conflict of interest.

## Publisher's Note

All claims expressed in this article are solely those of the authors and do not necessarily represent those of their affiliated organizations, or those of the publisher, the editors and the reviewers. Any product that may be evaluated in this article, or claim that may be made by its manufacturer, is not guaranteed or endorsed by the publisher.
